# Supplementary material for: Longitudinal changes in IgG levels among COVID-19 recovered patients: A prospective cohort study
Source: PLoS One. 2021 Jun 11;16(6):e0251159. doi: 10.1371/journal.pone.0251159 (PMC8195379; doi:10.1371/journal.pone.0251159)
Supplement: S1 File — (DOCX) [file pone.0251159.s001.docx]

Longitudinal changes in IgG levels among COVID-19 recovered patients: A prospective cohort study.

Supplementary material

**Liaison SARS CoV-2 S1S2 IgG Method Performance Verification**

The method for quantitative determination of IgG anti-S1 and IgG anti-S2 specific antibodies to SARS-CoV-2 is an indirect chemiluminescence immunoassay (CLIA). The specific recombinant S1 and S2 antigens are used for coating magnetic particles (solid phase) and mouse monoclonal antibodies to human IgG are linked to an isoluminol derivative (isoluminolantibody conjugate).

During the first incubation, the SARS-CoV-2 IgG antibodies present in calibrators, samples or controls bind to the solid phase through the recombinant S1 and S2 antigens. During the second incubation the antibody conjugate reacts with IgG to SARS-CoV-2 already bound to the solid phase. After each incubation, the unbound material is removed with a wash cycle. Subsequently, the starter reagents are added and a flash chemiluminescence reaction is thus induced. The light signal, and hence the amount of isoluminol-antibody conjugate, is measured by a photomultiplier as relative light units (RLU) and is indicative of IgG to SARS-CoV2 concentration present in calibrators, samples or controls.

**INTRODUCTION**

**Protocols used by ZMH Core laboratory to verify method performance**

**P**erformance verification studies are part of the installation of a new instrument, or the addition of a new assay to an existing instrument. The purpose of these studies is documenting that the performance of the new assay being installed is consistent with product claims as stated in the manufacturer’s product labeling and following ZMHAD clinical laboratory regulations and accreditation requirements.

The protocol used by ZMH Core lab to verify LIAISON SARS CoV-2 S1S2 IgG reagent performance specifications have been designed as per Clinical and Laboratory Standards Institute (CLSI) as follows:

Before reporting patient test results, the assay should:

(i) Demonstrate that it can obtain performance specifications comparable to those established by the manufacturer for the following performance characteristics: (A) Accuracy , (B) Precision , (C) Reportable range of test results for the test system

(ii) Verify that the manufacturer's reference intervals (normal values) are appropriate for the laboratory's patient population.

1. **Precision**

Following the CLSI document EP5-A3. A five-day precision study was performed by using Negative and positive control samples. Samples were tested with the LIAISON® SARS-CoV-2 S1/S2 IgG assay in 5 replicates per run, 3 runs per day for five operating days on one LIAISON® XL Analyzer. The Observed Coefficient of variation % ranged from 0.01 % in the negative control samples to 1.7 % in the positive control samples.

1. **Linearity:**

Linearity was evaluated according to CLSI EP6-A. One sample was tested neat and after dilutions with the specimen diluent. The results were analyzed as a linear regression of the Expected vs. Observed values. The resulting regression equation is: Observed = 1.043 (Expected) - 0.2807; R^2^= 0.997

1. **Analytical sensitivity**

Following CLSI EP17-A2: Analytical sensitivity is defined as the minimum detectable dose distinguishable from zero by 1.654 standard deviations. The limit of detection (LoD) for the LIAISON® SARS-CoV-2 S1/S2 IgG assay was verified; 3.8 AU/mL.

1. **Diagnostic sensitivity**

The sensitivity was determined by investigating 50 patient’s samples collected over the course of time. Infection with SARS-CoV-2 was confirmed by RT-PCR test at the time of the diagnosis. The LIAISON® SARS-CoV-2 S1/S2 IgG test was performed on samples collected at the time of admission and thereafter up.

The diagnostic sensitivity was evaluated in three groups, i.e. the early samples (≤ 5 days after diagnosis), the samples between 5 and 15 days after diagnosis, and the later samples (> 15 days after diagnosis).

|  | LIAISON® SARS-CoV-2 S1/S2 IgG | | | Total | Sensitivity (Wilson 95% CI) |
| --- | --- | --- | --- | --- | --- |
|  | < 12 AU/mL | 12-15 AU/mL | ≥ 15 AU/mL |  |  |
| ≤ 5 days | 11 | 1 | 4 | 16 | 24.9% (14.0%-39.4%) |
| 5-15 days | 1 | 1 | 12 | 14 | 90.2% (79.2%-95.8%) |
| ≥ 15 days | 0 | 1 | 19 | 20 | 97.3% (86.8%-99.5%) |

1. **Diagnostic specificity**

One hundred presumed SARS-CoV-2 negative samples collected before the COVID19 outbreak (80 fresh frozen plasma (FFP) samples from blood donors, 10 stored HIV positive samples, and 10 stored Anti-nuclear autoantibodies (ANA) positive patient’s samples) were tested resulting in 99.25% clinical specificity (95% CI: 98.5% – 100%). The following table shows the results:

|  | LIAISON® SARS-CoV-2 S1/S2 IgG | | | Total | Specificity (Wilson 95% CI) |
| --- | --- | --- | --- | --- | --- |
|  | < 12 AU/mL | 12-15 AU/mL | ≥ 15 AU/mL |  |  |
| Pre pandemic FFP samples | 78 | 1 | 1 | 80 | 98.5% (97.5% - 99.2%) |
| Pre pandemic HIV positive samples | 10 | 0 | 0 | 10 | 100 % |
| Pre pandemic ANA positive samples | 10 | 0 | 0 | 10 | 100% |

After method verification studies completed, we added the SARS-CoV-2 S1/S2 IgG test to our test menu and attached the following interpretation to test results:

**INTERPRETATION**

This assay was performed using Liaison XL SARS CoV-2 S1/S2 IgG reagent, which uses Chemiluminescence Immune Assay Technology. For this test, correlation with epidemiological risk factors and other lab findings is recommended. Serological results should not be used as a sole basis to diagnose or exclude recent or passed SARS CoV-2 infection.

- **0 – 12 AU/mL: Negative result:**

Sample does not contain SARS CoV-2 S1/S2 IgG antibodies.

The negative result does not rule out SARS CoV-2 infection

- **12 - 15 AU/mL: Equivocal result:**

This result should be interpreted with care, as it may indicate a low level of IgG antibodies.

- **More than 15 AU/mL: Positive result:**

Result suggest recent or prior infection with SARS CoV-2.

Protective immunity cannot be inferred based on this result.

Infrequently, false positive results may be due to prior infection with other Coronaviruses.

**Table S1: BSTI scoring patterns for COVID-19 pneumonia**

| Pattern | Description |
| --- | --- |
| Classic COVID-19  (100% confidence for COVID-19 pneumonia | Lower lobe predominant, peripheral predominant, multiple, bilateral ground-glass opacities of GGO +/-   - Crazy-paving - Peripheral consolidation (organising pneumonia patterns) - Air Bronchograms - Reverse halo/perilobular pattern |
| Probable COVID-19  (71-99% confidence for COVID-19 pneumonia) | - Lower lobe predominant mix of bronchocentric and peripheral consolidation. - Reverse halo/perilobular pattern - GGO scarce |
| Indeterminate  (<70% confidence for COVID-19 pneumonia) | - Does not fit to definite, probable or non-COVID - Manifests above patterns, but the clinical context is wrong, or suggests an alternative diagnosis (eg an interstitial lung disease in a connective tissue disease setting) |
| Non-COVID-19 | - Lobar pneumonia - Cavitating infections - Tree-in-bud/ centrilobular nodularity - Lymphadenopathy, effusions - Established pulmonary fibrosis |
| Normal | Normal examination |

**Table S2: Baseline laboratory and serology characteristics.**

|  | N | Mean (SD) | Median (IQR) | Min-Max |
| --- | --- | --- | --- | --- |
| Symptoms Duration | 113 | 3.72 (3.61) | 3 (1-5) | 0-14 |
| WBC | 113 | 6.17 (2.59) | 5.49 (4.74-6.94) | 2.36-17.7 |
| ALC | 113 | 1.67 (0.74) | 1.56 (1.16-2.13) | 0.21-4.5 |
| LDH | 113 | 227 (85.9) | 207 (180-258) | 1.2-779 |
| DDIMER | 107 | 0.437 (0.448) | 0.27 (0.27-0.46) | 0.02-4.3 |
| CPK | 113 | 180 (237) | 101 (75-182) | 0.3-1714 |
| Procalcitonin | 113 | 0.0489 (0.0552) | 0.03 (0.02-0.05) | 0.01-0.37 |
| Ferritin | 112 | 365 (524) | 212 (130-369) | 16.5-3913 |
| CRP | 113 | 18.4 (32.1) | 5.5 (1.9-16) | 0.9-177 |
| QTC1 | 111 | 423 (23.6) | 425 (407-437) | 352-499 |
| QTC2 | 93 | 430 (20.7) | 432 (416-443) | 382-489 |
| QTC3 | 92 | 427 (21.9) | 425 (412-440) | 375-498 |
| Viral shedding (days) | 106 | 12.5 (7.03) | 12 (8-16) | 1-35 |
| Treatment Duration | 112 | 5.89 (2.31) | 7 (5-7) | 0-16 |
|  |  |  |  |  |
| Timing of first visit (days) | 113 | 18 (14.9) | 14 (10-19) | 2-88 |
| First visit IgG level | 113 | 71.5 (90) | 42.7 (11.5-83.2) | 3.5-401 |
| Timing of second visit (days) | 63 | 53.1 (22) | 51 (42-70) | 10-105 |
| Second visit IgG level | 63 | 114 (100) | 88.8 (43-141) | 6.61-401 |
| Timing of third visit (days) | 27 | 108 (36.3) | 121 (59-133) | 49-168 |
| Third visit IgG level | 27 | 106 (95.4) | 81.2 (42.6-127) | 17-401 |

**Table S3: Demographic and clinical characteristics of the patients by IgG response during visit 1 and visit 2**

|  | IgG visit 1 | | |  | IgG visit 2 | | |
| --- | --- | --- | --- | --- | --- | --- | --- |
|  | **Negative** | **Positive** | **Total** |  | **Negative** | **Positive** | **Total** |
| Age |  |  |  |  |  |  |  |
| *20-29* | 5 (55.56) | 4 (44.44) | 9 (100) |  | 1 (33.33) | 2 (66.67) | 3 (100) |
| *30-39* | 12 (41.38) | 17 (58.62) | 29 (100) |  | 2 (13.33) | 13 (86.67) | 15 (100) |
| *40-49* | 9 (32.14) | 19 (67.86) | 28 (100) |  | 3 (16.67) | 15 (83.33) | 18 (100) |
| *50-59* | 8 (26.67) | 22 (73.33) | 30 (100) |  | 0 (0) | 17 (100) | 17 (100) |
| *60+* | 2 (11.76) | 15 (88.24) | 17 (100) |  | 0 (0) | 10 (100) | 10 (100) |
| Gender |  |  |  |  |  |  |  |
| *Women* | 4 (28.57) | 10 (71.43) | 14 (100) |  | 0 (0) | 8 (100) | 8 (100) |
| *Men* | 32 (32.32) | 67 (67.68) | 99 (100) |  | 6 (10.91) | 49 (89.09) | 55 (100) |
| Nationality |  |  |  |  |  |  |  |
| *Emirati National* | 17 (40.48) | 25 (59.52) | 42 (100) |  | 1 (5.56) | 17 (94.44) | 18 (100) |
| *Non-National* | 19 (27.14) | 51 (72.86) | 70 (100) |  | 4 (9.09) | 40 (90.91) | 44 (100) |
| Case status |  |  |  |  |  |  |  |
| *Confirmed* | 31 (32.29) | 65 (67.71) | 69 (100) |  | 5 (9.43) | 48 (90.57) | 53 (100) |
| *Suspected* | 5 (29.41) | 12 (70.59) | 17 (100) |  | 1 (10) | 9 (90) | 10 (100) |
| Symptoms |  |  |  |  |  |  |  |
| *No* | 10 (41.67) | 14 (58.33) | 24 (100) |  | 1 (5.88) | 16 (94.12) | 17 (100) |
| *Yes* | 26 (29.21) | 63 (70.79) | 89 (100) |  | 5 (10.87) | 41 (89.13) | 46 (100) |
| ICU/HDU |  |  |  |  |  |  |  |
| *No* | 36 (33.03) | 73 (66.97) | 109 (100) |  | 6 (10.17) | 53 (89.83) | 59 (100) |
| *Yes* | 0 (0) | 4 (100) | 4 (100) |  | 0 (0) | 4 (100) | 4 (100) |
| Hypoxia |  |  |  |  |  |  |  |
| *No* | 34 (33.66) | 67 (66.34) | 101 (100) |  | 6 (11.11) | 48 (88.89) | 54 (100) |
| *Yes* | 2 (16.67) | 10 (83.33) | 12 (100) |  | 0 (0) | 9 (100) | 9 (100) |
| Diabetes Mellitus |  |  |  |  |  |  |  |
| *No* | 31 (35.23) | 57 (64.77) | 88 (100) |  | 6 (12.5) | 42 (87.5) | 48 (100) |
| *Yes* | 5 (20) | 20 (80) | 25 (100) |  | 0 (0) | 15 (100) | 15 (100) |
| Hypertension |  |  |  |  |  |  |  |
| *No* | 28 (34.57) | 53 (65.43) | 81 (100) |  | 5 (11.9) | 37 (88.1) | 42 (100) |
| *Yes* | 8 (25) | 24 (75) | 32 (100) |  | 1 (4.76) | 20 (95.24) | 21 (100) |
| Heart Disease |  |  |  |  |  |  |  |
| *No* | 33 (31.73) | 71 (68.27) | 104 (100) |  | 5 (8.62) | 53 (91.38) | 58 (100) |
| *Yes* | 3 (33.33) | 6 (66.67) | 9 (100) |  | 1 (20) | 4 (80) | 5 (100) |
| Renal Disease |  |  |  |  |  |  |  |
| *No* | 36 (33.33) | 72 (66.67) | 108 (100) |  | 6 (10) | 54 (90) | 60 (100) |
| *Yes* | 0 (0) | 5 (100) | 5 (100) |  | 0 (0) | 3 (100) | 3 (100) |
| Respiratory Disease |  |  |  |  |  |  |  |
| *No* | 36 (33.03) | 73 (66.97) | 109 (100) |  | 6 (9.84) | 55 (90.16) | 61 (100) |
| *Yes* | 0 (0) | 4 (100) | 4 (100) |  | 0 (0) | 2 (100) | 2 (100) |
| Immune Disease |  |  |  |  |  |  |  |
| *No* | 35 (31.25) | 77 (68.75) | 112 (100) |  | 6 (9.52) | 57 (90.48) | 63 (100) |
| *Yes* | 1 (100) | 0 (0) | 1 (100) |  | 0 (0) | 0 (0) | 0 (0) |
| URTI |  |  |  |  |  |  |  |
| *No* | 20 (24.69) | 61 (75.31) | 81 (100) |  | 3 (6.12) | 46 (93.88) | 49 (100) |
| *Yes* | 13 (50) | 13 (50) | 26 (100) |  | 3 (23.08) | 10 (76.92) | 13 (100) |
| Fever |  |  |  |  |  |  |  |
| *No* | 14 (30.43) | 32 (69.57) | 46 (100) |  | 4 (16.67) | 20 (83.33) | 24 (100) |
| *Yes* | 19 (31.15) | 42 (68.85) | 61 (100) |  | 2 (5.26) | 36 (94.74) | 38 (100) |
| Cough |  |  |  |  |  |  |  |
| *No* | 17 (34.69) | 32 (65.31) | 49 (100) |  | 4 (13.33) | 26 (86.67) | 30 (100) |
| *Yes* | 16 (27.59) | 42 (71.41) | 58 (100) |  | 2 (6.25) | 30 (93.75) | 32 (100) |
| SOB |  |  |  |  |  |  |  |
| *No* | 29 (30.77) | 65 (69.15) | 94 (100) |  | 6 (10.71) | 50 (89.29) | 56 (100) |
| *Yes* | 4 (30.77) | 9 (69.23) | 13 (100) |  | 0 (0) | 6 (100) | 6 (100) |
| Fatigue |  |  |  |  |  |  |  |
| *No* | 23 (31.51) | 50 (68.49) | 73 (100) |  | 5 (11.9) | 37 (88.1) | 42 (100) |
| *Yes* | 10 (29.41) | 24 (70.59) | 34 (100) |  | 1 (5) | 19 (95) | 20 (100) |
| Loss of taste |  |  |  |  |  |  |  |
| *No* | 27 (29.35) | 65 (70.65) | 92 (100) |  | 6 (10.71) | 50 (89.29) | 56 (100) |
| *Yes* | 6 (40) | 9 (60) | 15 (100) |  | 0 (0) | 6 (100) | 6 (100) |
| Loss of smell |  |  |  |  |  |  |  |
| *No* | 26 (28.26) | 66 (71.74) | 92 (100) |  | 6 (10.34) | 52 (89.66) | 58 (100) |
| *Yes* | 7 (46.67) | 8 (53.33) | 15 (100) |  | 0 (0) | 4 (100) | 4 (100) |
| Sore throat |  |  |  |  |  |  |  |
| *No* | 23 (28.4) | 58 (71.6) | 81 (100) |  | 4 (8.89) | 41 (91.11) | 45 (100) |
| *Yes* | 10 (38.46) | 16 (61.54) | 26 (100) |  | 2 (11.76) | 15 (88.24) | 17 (100) |
| Diarrhea/Nausea |  |  |  |  |  |  |  |
| *No* | 31 (31.63) | 67 (68.37) | 98 (100) |  | 6 (10.53) | 51 (89.47) | 57 (100) |
| *Yes* | 2 (22.22) | 7 (77.78) | 9 (100) |  | 0 (0) | 5 (100) | 5 (100) |
| Smoking |  |  |  |  |  |  |  |
| *No* | 35 (31.82) | 75 (68.18) | 110 (100) |  | 6 (9.68) | 56 (90.32) | 62 (100) |
| *Yes* | 1 (33.33) | 2 (66.67) | 3 (100) |  | 0 (0) | 1 (100) | 1 (100) |
| PCR |  |  |  |  |  |  |  |
| *Negative* | 2 (25) | 6 (75) | 8 (100) |  | 0 (0) | 5 (100) | 5 (100) |
| *Positive* | 34 (32.38) | 71 (67.62) | 105 (100) |  | 6 (10.34) | 52 (89.66) | 58 (100) |
| CT |  |  |  |  |  |  |  |
| *Negative* | 5 (71.43) | 2 (28.57) | 7 (100) |  | 0 (0) | 4 (100) | 4 (100) |
| *Positive* | 30 (28.85) | 74 (71.15) | 104 (100) |  | 6 (10.34) | 52 (89.66) | 58 (100) |
| Hydroxychloroquine |  |  |  |  |  |  |  |
| *No* | 12 (54.55) | 10 (45.45) | 22 (100) |  | 1 (25) | 3 (75) | 4 (100) |
| *Yes* | 24 (26.37) | 67 (73.63) | 91 (100) |  | 5 (8.47) | 54 (91.53) | 59 (100) |
| Favipiravir |  |  |  |  |  |  |  |
| *No* | 24 (51.06) | 23 (48.94) | 47 (100) |  | 6 (26.09) | 17 (73.91) | 23 (100) |
| *Yes* | 12 (18.18) | 54 (81.82) | 66 (100) |  | 0 (0) | 40 (100) | 40 (100) |
| Kelatra (Lopinavir and Ritonavir) |  |  |  |  |  |  |  |
| *No* | 35 (32.71) | 72 (67.29) | 107 (100) |  | 6 (10.34) | 52 (89.66) | 58 (100) |
| *Yes* | 1 (16.67) | 5 (83.33) | 6 (100) |  | 0 (0) | 5 (100) | 5 (100) |

**Table S4: Baseline age, laboratory and serology characteristics of patients by IgG response during visit 1**

|  | Negative for IgG | | | | Positive for IgG | | | |
| --- | --- | --- | --- | --- | --- | --- | --- | --- |
|  | **N** | **Mean (SD)** | **Median (IQR)** | **Min-Max** | **N** | **Mean (SD)** | **Median (IQR)** | **Min-Max** |
| Age^*^ | 36 | 41.9 (12) | 40 (33-50.5) | 20-66 | 77 | 47.8 (11.4) | 45 (39-57) | 26-67 |
| Symptoms Duration | 36 | 2.81 (3) | 2 (0-4) | 0-14 | 77 | 4.14 (3.81) | 3 (1-5) | 0-14 |
| WBC | 36 | 6.47 (2.73) | 5.9 (5.02-6.63) | 2.85-17.7 | 77 | 6.03 (2.52) | 5.22 (4.68-7.08) | 2.36-17.6 |
| ALC | 36 | 1.76 (0.753) | 1.79 (1.36-2.09) | 0.21-3.83 | 77 | 1.63 (0.735) | 1.52 (1.12-2.14) | 0.28-4.5 |
| LDH^*^ | 36 | 198 (63.3) | 193 (174-216) | 1.2-379 | 77 | 241 (91.7) | 223 (186-266) | 126-779 |
| DDIMER | 34 | 0.359 (0.256) | 0.27 (0.27-0.36) | 0.02-1.65 | 73 | 0.473 (0.511) | 0.28 (0.27-0.47) | 0.22-4.3 |
| CPK | 36 | 170 (206) | 96.5 (69.5-187) | 0.3-1105 | 77 | 185 (252) | 105 (81-182) | 0.4-1714 |
| Procalcitonin | 36 | 0.0506 (0.0658) | 0.03 (0.02-0.05) | 0.01-0.37 | 77 | 0.0482 (0.0499) | 0.03 (0.02-0.06) | 0.01-0.3 |
| Ferritin^*^ | 36 | 197 (119) | 171 (119-239) | 18.7-518 | 76 | 445 (616) | 239 (143-470) | 16.5-3913 |
| CRP^*^ | 36 | 11.7 (27.7) | 4 (1.25-8.25) | 0.9-159 | 77 | 21.6 (33.6) | 7.5 (1.9-23.8) | 0.9-177 |
| QTC1 | 36 | 423 (24.2) | 424 (406-434) | 386-499 | 75 | 423 (23.5) | 426 (408-440) | 352-482 |
| QTC2 | 24 | 437 (24.8) | 439 (414-449) | 395-489 | 69 | 427 (18.7) | 429 (417-440) | 382-468 |
| QTC3 | 25 | 433 (24.7) | 425 (415-446) | 392-498 | 67 | 424 (20.5) | 425 (411-437) | 375-469 |
| Viral shedding (days) | 33 | 13.2 (8.35) | 14 (8-16) | 1-35 | 73 | 12.2 (6.38) | 12 (8-15) | 1-30 |
| Treatment Duration^*^ | 35 | 4.83 (3.26) | 5 (4-7) | 0-16 | 77 | 6.38 (1.51) | 7 (7-7) | 0-10 |
|  |  |  |  |  |  |  |  |  |
| Timing of first visit (days) | 36 | 11.8 (10) | 10 (6.5-13.5) | 2-58 | 77 | 20.8 (16) | 16 (12-21) | 3-88 |
| First visit IgG level | 36 | 7.91 (3.92) | 7.64 (3.93-11) | 3.5-14.9 | 77 | 101 (95.4) | 61 (39.8-124) | 15.1-401 |
| Timing of second visit (days) | 17 | 39.8 (22.4) | 38 (18-48) | 10-92 | 46 | 58 (20) | 52.5 (47-71) | 10-105 |
| Second visit IgG level | 17 | 42.4 (34.8) | 29.1 (16.7-61.9) | 6.61-129 | 46 | 140 (104) | 117 (65.8-176) | 8.58-401 |
| Timing of third visit (days) | 7 | 74.6 (29.5) | 59 (53-114) | 49-119 | 20 | 120 (31) | 129 (117-139) | 49-168 |
| Third visit IgG level | 7 | 116 (132) | 49.8 (38.1-138) | 29.1-401 | 20 | 102 (83.1) | 84.7 (46.5-127) | 17-350 |

^*^ significant difference between the two groups (all p-values <0.05)

**Table S5: Baseline age, laboratory and serology characteristics of patients by IgG response during visit 2**

|  | Negative for IgG | | | | Positive for IgG | | | |
| --- | --- | --- | --- | --- | --- | --- | --- | --- |
|  | **N** | **Mean (SD)** | **Median (IQR)** | **Min-Max** | **N** | **Mean (SD)** | **Median (IQR)** | **Min-Max** |
| Age^*^ | 6 | 37.2 (8.33) | 38 (30-43) | 26-48 | 57 | 48.4 (11) | 49 (39-57) | 26-67 |
| Symptoms Duration | 6 | 3.83 (5.42) | 1 (1-6) | 0-14 | 57 | 3.12 (3.46) | 2 (0-4) | 0-14 |
| WBC | 6 | 5.89 (1.53) | 6.13 (4.22-6.63) | 4.09-8.11 | 57 | 6.03 (2.45) | 5.25 (4.73-6.9) | 2.64-17.6 |
| ALC^*^ | 6 | 2.3 (0.862) | 2.47 (2.26-2.95) | 0.65-2.98 | 57 | 1.57 (0.774) | 1.46 (1.12-1.92) | 0.21-4.5 |
| LDH | 6 | 191 (22.5) | 186 (179-204) | 164-229 | 57 | 230 (67.2) | 210 (185-265) | 126-478 |
| DDIMER | 5 | 1.19 (1.75) | 0.43 (0.27-0.69) | 0.27-4.3 | 52 | 0.367 (0.184) | 0.27 (0.27-0.425) | 0.02-1.11 |
| CPK | 6 | 167 (122) | 106 (98-211) | 85-397 | 57 | 197 (269) | 118 (71-198) | 44-1714 |
| Procalcitonin | 6 | 0.0483 (0.0652) | 0.02 (0.02-0.04) | 0.01-0.18 | 57 | 0.0553 (0.0677) | 0.03 (0.02-0.06) | 0.01-0.37 |
| Ferritin | 6 | 207 (102) | 208 (104-310) | 93-322 | 56 | 403 (520) | 245 (136-438) | 16.5-2796 |
| CRP | 6 | 3.28 (3.25) | 1.85 (1.6-3.9) | 0.9-9.6 | 57 | 17.8 (25.9) | 6.6 (1.9-20.4) | 0.9-111 |
| QTC1 | 6 | 410 (26.1) | 405 (391-419) | 386-456 | 57 | 422 (20) | 425 (408-435) | 375-467 |
| QTC2^*^ | 4 | 405 (15.6) | 400 (394-416) | 393-427 | 54 | 426 (18.8) | 426 (416-439) | 382-477 |
| QTC3 | 4 | 417 (25.4) | 414 (397-437) | 392-449 | 54 | 426 (21.5) | 424 (411-440) | 379-479 |
| Viral shedding (days) | 6 | 14.2 (3.6) | 14 (12-17) | 9-19 | 55 | 13.1 (6.43) | 13 (9-17) | 1-30 |
| Treatment Duration^*^ | 6 | 4.5 (2.35) | 5 (5-5) | 0-7 | 57 | 6.44 (1.35) | 7 (7-7) | 0-8 |
|  |  |  |  |  |  |  |  |  |
| Timing of first visit (days) | 6 | 17 (21.7) | 10.5 (3-16) | 2-60 | 57 | 15.6 (7.97) | 15 (10-19) | 3-57 |
| First visit IgG level | 6 | 14.5 (7.12) | 15.7 (7.9-18.3) | 4.93-24.5 | 57 | 75.7 (94.9) | 44.2 (15.1-81.1) | 3.5-401 |
| Timing of second visit (days) | 6 | 44.2 (28.6) | 46 (16-55) | 12-90 | 57 | 54 (21.3) | 51 (43-70) | 10-105 |
| Second visit IgG level | 6 | 9.4 (2.47) | 8.86 (7.45-11.5) | 6.61-13.1 | 57 | 125 (99) | 101 (58.4-145) | 12.6-401 |
| Timing of third visit (days) | 3 | 115 (60.7) | 129 (49-168) | 49-168 | 24 | 107 (34) | 120 (65-132) | 49-146 |
| Third visit IgG level | 3 | 28.1 (18.8) | 17.4 (17-49.8) | 17-49.8 | 24 | 115 (96.9) | 97.1 (48.6-133) | 26.7-401 |

^*^ significant difference between the two groups (all p-values <0.05)

**Table S6: Baseline age, laboratory and serology characteristics of patients by IgG response during visit 3 (all patients tested positive)**

|  | Positive for IgG | | | |
| --- | --- | --- | --- | --- |
|  | **N** | **Mean (SD)** | **Median (IQR)** | **Min-Max** |
| Age | 27 | 48.3 (11.3) | 48 (39-58) | 26-66 |
| Symptoms Duration | 27 | 3.15 (3.63) | 2 (1-4) | 0-14 |
| WBC | 27 | 6.02 (1.62) | 5.55 (4.94-7.15) | 3.35-9.34 |
| ALC | 27 | 1.75 (0.738) | 1.56 (1.2-2.29) | 0.65-3.83 |
| LDH | 27 | 245 (70.4) | 232 (185-291) | 162-478 |
| DDIMER | 22 | 0.432 (0.228) | 0.34 (0.27-0.51) | 0.27-1.11 |
| CPK | 27 | 244 (323) | 121 (95-309) | 60-1714 |
| Procalcitonin | 27 | 0.0689 (0.0926) | 0.03 (0.02-0.06) | 0.01-0.37 |
| Ferritin | 27 | 363 (359) | 221 (140-450) | 93-1621 |
| CRP | 27 | 15.2 (22.7) | 6.6 (2.5-14.8) | 0.9-100 |
| QTC1 | 27 | 424 (21.5) | 427 (400-436) | 386-467 |
| QTC2 | 27 | 426 (20.5) | 424 (416-443) | 393-477 |
| QTC3 | 27 | 426 (21.1) | 416 (411-441) | 392-479 |
| Viral shedding (days) | 27 | 13.6 (5.33) | 13 (10-17) | 2-28 |
| Treatment Duration | 27 | 6.26 (1.29) | 7 (5-7) | 2-7 |
| Timing of first visit (days) | 27 | 15.7 (11.2) | 15 (9-19) | 3-60 |
|  |  |  |  |  |
| First visit IgG level | 27 | 68.4 (94.9) | 44.1 (11.5-61) | 3.5-401 |
| Timing of second visit (days) | 27 | 46.6 (23.3) | 48 (28-57) | 10-96 |
| Second visit IgG level | 27 | 104 (90.7) | 85.5 (38.1-129) | 7.45-334 |
| Timing of third visit (days) | 27 | 108 (36.3) | 121 (59-133) | 49-168 |
| Third visit IgG level | 27 | 106 (95.4) | 81.2 (42.6-127) | 17-401 |

**Table S7: Demographic and clinical characteristics of the patients by IgG level change between visits**

|  | Visit 1 to visit 2 | | |  | Visit 2 to visit 3 | | |  | Visit 1 to visit 3 | | |
| --- | --- | --- | --- | --- | --- | --- | --- | --- | --- | --- | --- |
|  | **Decline** | **Incline** | **Total** |  | **Decline** | **Incline** | **Total** |  | **Decline** | **Incline** | **Total** |
| Age |  |  |  |  |  |  |  |  |  |  |  |
| *20-29* | 1 (33.33) | 2 (66.67) | 3 (100) |  | 0 (0) | 1 (100) | 1 (100) |  | 0 (0) | 1 (100) | 1 (100) |
| *30-39* | 4 (26.67) | 11 (73.33) | 15 (100) |  | 2 (28.57) | 5 (71.43) | 7 (100) |  | 3 (42.86) | 4 (57.14) | 7 (100) |
| *40-49* | 5 (27.78) | 13 (72.22) | 18 (100) |  | 5 (71.43) | 2 (28.57) | 7 (100) |  | 2 (28.57) | 5 (71.43) | 7 (100) |
| *50-59* | 1 (5.88) | 16 (94.12) | 17 (100) |  | 3 (42.86) | 4 (57.14) | 7 (100) |  | 3 (42.86) | 4 (57.14) | 7 (100) |
| *60+* | 0 (0) | 10 (100) | 10 (100) |  | 2 (40) | 3 (60) | 5 (100) |  | 0 (0) | 5 (100) | 5 (100) |
| Gender |  |  |  |  |  |  |  |  |  |  |  |
| *Women* | 0 (0) | 8 (100) | 8 (100) |  | 2 (50) | 2 (50) | 4 (100) |  | 1 (25) | 3 (75) | 4 (100) |
| *Men* | 11 (20) | 44 (80) | 55 (100) |  | 10 (43.48) | 13 (56.52) | 23 (100) |  | 7 (30.43) | 16 (69.57) | 23 (100) |
| Nationality |  |  |  |  |  |  |  |  |  |  |  |
| *Emirati National* | 6 (33.33) | 12 (66.67) | 18 (100) |  | 3 (75) | 1 (25) | 4 (100) |  | 4 (100) | 0 (0) | 4 (100) |
| *Non-National* | 4 (9.09) | 40 (90.91) | 44 (100) |  | 9 (40.91) | 13 (59.09) | 22 (100) |  | 4 (18.18) | 18 (81.82) | 22 (100) |
| Case status |  |  |  |  |  |  |  |  |  |  |  |
| *Confirmed* | 9 (16.98) | 44 (83.02) | 53 (100) |  | 10 (45.45) | 12 (54.55) | 22 (100) |  | 7 (31.82) | 15 (68.18) | 22 (100) |
| *Suspected* | 2 (20) | 8 (80) | 10 (100) |  | 2 (40) | 3 (60) | 5 (100) |  | 1 (20) | 4 (80) | 5 (100) |
| Symptoms |  |  |  |  |  |  |  |  |  |  |  |
| *No* | 4 (23.53) | 13 (76.47) | 17 (100) |  | 2 (33.33) | 4 (66.67) | 6 (100) |  | 1 (16.67) | 5 (83.33) | 6 (100) |
| *Yes* | 7 (15.22) | 39 (84.78) | 46 (100) |  | 10 (47.62) | 11 (52.38) | 21 (100) |  | 7 (33.33) | 14 (66.67) | 21 (100) |
| ICU/HDU |  |  |  |  |  |  |  |  |  |  |  |
| *No* | 11 (18.64) | 48 (81.36) | 59 (100) |  | 12 (46.15) | 14 (53.85) | 26 (100) |  | 8 (30.77) | 18 (69.23) | 26 (100) |
| *Yes* | 0 (0) | 4 (100) | 4 (100) |  | 0 (0) | 1 (100) | 1 (100) |  | 0 (0) | 1 (100) | 1 (100) |
| Hypoxia |  |  |  |  |  |  |  |  |  |  |  |
| *No* | 10 (18.52) | 44 (81.48) | 54 (100) |  | 12 (50) | 12 (50) | 24 (100) |  | 7 (29.17) | 17 (70.83) | 24 (100) |
| *Yes* | 1 (11.11) | 8 (88.89) | 9 (100) |  | 0 (0) | 3 (100) | 3 (100) |  | 1 (33.33) | 2 (66.67) | 3 (100) |
| Diabetes Mellitus |  |  |  |  |  |  |  |  |  |  |  |
| *No* | 9 (18.75) | 39 (81.25) | 48 (100) |  | 10 (50) | 10 (50) | 20 (100) |  | 6 (30) | 14 (70) | 20 (100) |
| *Yes* | 2 (13.33) | 13 (86.67) | 15 (100) |  | 2 (28.57) | 5 (71.43) | 7 (100) |  | 2 (28.57) | 5 (71.43) | 7 (100) |
| Hypertension |  |  |  |  |  |  |  |  |  |  |  |
| *No* | 8 (19.05) | 34 (80.95) | 42 (100) |  | 8 (47.06) | 9 (52.94) | 17 (100) |  | 5 (29.41) | 12 (70.59) | 17 (100) |
| *Yes* | 3 (14.29) | 18 (85.71) | 21 (100) |  | 4 (40) | 6 (60) | 10 (100) |  | 3 (30) | 7 (70) | 10 (100) |
| Heart Disease |  |  |  |  |  |  |  |  |  |  |  |
| *No* | 10 (17.24) | 48 (82.76) | 58 (100) |  | 11 (45.83) | 13 (54.17) | 24 (100) |  | 7 (29.17) | 17 (70.83) | 24 (100) |
| *Yes* | 1 (20) | 4 (80) | 5 (100) |  | 1 (33.33) | 2 (66.67) | 3 (100) |  | 1 (33.33) | 2 (66.67) | 3 (100) |
| Renal Disease |  |  |  |  |  |  |  |  |  |  |  |
| *No* | 11 (18.33) | 49 (81.67) | 60 (100) |  | 11 (44) | 14 (56) | 25 (100) |  | 8 (32) | 17 (68) | 25 (100) |
| *Yes* | 0 (0) | 3 (100) | 3 (100) |  | 1 (50) | 1 (50) | 2 (100) |  | 0 (0) | 2 (100) | 2 (100) |
| Respiratory Disease |  |  |  |  |  |  |  |  |  |  |  |
| *No* | 11 (18.03) | 50 (81.97) | 61 (100) |  | 12 (46.15) | 14 (53.85) | 26 (100) |  | 8 (30.77) | 18 (69.23) | 26 (100) |
| *Yes* | 0 (0) | 2 (100) | 2 (100) |  | 0 (0) | 1 (100) | 1 (100) |  | 0 (0) | 1 (100) | 1 (100) |
| Immune Disease |  |  |  |  |  |  |  |  |  |  |  |
| *No* | 11 (17.46) | 52 (82.54) | 63 (100) |  | 12 (44.44) | 15 (55.56) | 27 (100) |  | 8 (29.63) | 19 (70.37) | 27 (100) |
| *Yes* | 0 (0) | 0 (0) | 0 (0) |  | 0 (0) | 0 (0) | 0 (0) |  | 0 (0) | 0 (0) | 0 (0) |
| URTI |  |  |  |  |  |  |  |  |  |  |  |
| *No* | 9 (18.37) | 40 (81.63) | 49 (100) |  | 11 (55) | 9 (45) | 20 (100) |  | 6 (30) | 14 (70) | 20 (100) |
| *Yes* | 2 (15.38) | 11 (84.62) | 13 (100) |  | 1 (14.29) | 6 (85.71) | 7 (100) |  | 2 (28.57) | 5 (71.43) | 7 (100) |
| Fever |  |  |  |  |  |  |  |  |  |  |  |
| *No* | 6 (25) | 18 (75) | 24 (100) |  | 6 (54.55) | 5 (45.45) | 11 (100) |  | 3 (27.27) | 8 (72.73) | 11 (100) |
| *Yes* | 5 (13.16) | 33 (86.84) | 38 (100) |  | 6 (37.5) | 10 (62.5) | 16 (100) |  | 5 (31.25) | 11 (68.75) | 16 (100) |
| Cough |  |  |  |  |  |  |  |  |  |  |  |
| *No* | 8 (26.67) | 22 (73.33) | 30 (100) |  | 7 (58.33) | 5 (41.67) | 12 (100) |  | 5 (41.67) | 7 (58.33) | 12 (100) |
| *Yes* | 3 (9.38) | 29 (90.63) | 32 (100) |  | 5 (33.33) | 10 (66.67) | 15 (100) |  | 3 (20) | 12 (80) | 15 (100) |
| SOB |  |  |  |  |  |  |  |  |  |  |  |
| *No* | 11 (19.64) | 45 (80.36) | 56 (100) |  | 11 (47.83) | 12 (52.17) | 23 (100) |  | 8 (34.78) | 15 (65.22) | 23 (100) |
| *Yes* | 0 (0) | 6 (100) | 6 (100) |  | 1 (25) | 3 (75) | 4 (100) |  | 0 (0) | 4 (100) | 4 (100) |
| Fatigue |  |  |  |  |  |  |  |  |  |  |  |
| *No* | 8 (19.05) | 34 (80.95) | 42 (100) |  | 7 (41.18) | 10 (58.82) | 17 (100) |  | 4 (23.53) | 13 (76.47) | 17 (100) |
| *Yes* | 3 (15) | 17 (85) | 20 (100) |  | 5 (50) | 5 (50) | 10 (100) |  | 4 (40) | 6 (60) | 10 (100) |
| Loss of taste |  |  |  |  |  |  |  |  |  |  |  |
| *No* | 11 (19.64) | 45 (80.36) | 56 (100) |  | 10 (43.48) | 13 (56.52) | 23 (100) |  | 8 (34.78) | 15 (65.22) | 23 (100) |
| *Yes* | 0 (0) | 6 (100) | 6 (100) |  | 2 (50) | 2 (50) | 4 (100) |  | 0 (0) | 4 (100) | 4 (100) |
| Loss of smell |  |  |  |  |  |  |  |  |  |  |  |
| *No* | 11 (18.97) | 47 (81.03) | 58 (100) |  | 11 (45.83) | 13 (54.17) | 24 (100) |  | 8 (33.33) | 16 (66.67) | 24 (100) |
| *Yes* | 0 (0) | 4 (100) | 4 (100) |  | 1 (33.33) | 2 (66.67) | 3 (100) |  | 0 (0) | 3 (100) | 3 (100) |
| Sore throat |  |  |  |  |  |  |  |  |  |  |  |
| *No* | 8 (17.78) | 37 (82.22) | 45 (100) |  | 10 (47.62) | 11 (52.38) | 21 (100) |  | 5 (23.81) | 16 (76.19) | 21 (100) |
| *Yes* | 3 (17.65) | 14 (82.35) | 17 (100) |  | 2 (33.33) | 4 (66.67) | 6 (100) |  | 3 (50) | 3 (50) | 6 (100) |
| Diarrhea/Nausea |  |  |  |  |  |  |  |  |  |  |  |
| *No* | 11 (19.3) | 46 (80.7) | 57 (100) |  | 11 (42.31) | 15 (57.69) | 26 (100) |  | 7 (26.92) | 19 (73.08) | 26 (100) |
| *Yes* | 0 (0) | 5 (100) | 5 (100) |  | 1 (100) | 0 (0) | 1 (100) |  | 1 (100) | 0 (0) | 1 (100) |
| Smoking |  |  |  |  |  |  |  |  |  |  |  |
| *No* | 11 (17.74) | 51 (82.26) | 62 (100) |  | 12 (46.15) | 14 (53.85) | 26 (100) |  | 8 (30.77) | 18 (69.23) | 26 (100) |
| *Yes* | 0 (0) | 1 (100) | 1 (100) |  | 0 (0) | 1 (100) | 1 (100) |  | 0 (0) | 1 (100) | 1 (100) |
| PCR |  |  |  |  |  |  |  |  |  |  |  |
| *Negative* | 1 (20) | 4 (80) | 5 (100) |  | 1 (50) | 1 (50) | 2 (100) |  | 0 (0) | 2 (100) | 2 (100) |
| *Positive* | 10 (17.24) | 48 (82.76) | 58 (100) |  | 11 (44) | 14 (56) | 25 (100) |  | 8 (32) | 17 (68) | 25 (100) |
| CT |  |  |  |  |  |  |  |  |  |  |  |
| *Negative* | 1 (25) | 3 (75) | 4 (100) |  | 1 (100) | 0 (0) | 1 (100) |  | 1 (100) | 0 (0) | 1 (100) |
| *Positive* | 10 (17.24) | 48 (82.76) | 58 (100) |  | 11 (42.31) | 15 (57.69) | 26 (100) |  | 7 (26.92) | 19 (73.08) | 26 (100) |
| Hydroxychloroquine |  |  |  |  |  |  |  |  |  |  |  |
| *No* | 1 (25) | 3 (75) | 4 (100) |  |  |  |  |  |  |  |  |
| *Yes* | 10 (16.95) | 49 (83.05) | 59 (100) |  | 12 (44.44) | 15 (55.56) | 27 (100) |  | 8 (29.63) | 19 (70.37) | 27 (100) |
| Favipiravir |  |  |  |  |  |  |  |  |  |  |  |
| *No* | 6 (26.09) | 17 (73.91) | 23 (100) |  | 4 (44.44) | 5 (55.56) | 9 (100) |  | 3 (33.33) | 6 (66.67) | 9 (100) |
| *Yes* | 5 (12.5) | 35 (87.5) | 40 (100) |  | 8 (44.44) | 10 (55.56) | 18 (100) |  | 5 (27.78) | 13 (72.22) | 18 (100) |
| Kelatra (Lopinavir and Ritonavir) |  |  |  |  |  |  |  |  |  |  |  |
| *No* | 11 (18.97) | 47 (81.03) | 58 (100) |  | 11 (42.31) | 15 (57.69) | 26 (100) |  | 8 (30.77) | 18 (69.23) | 26 (100) |
| *Yes* | 0 (0) | 5 (100) | 5 (100) |  | 1 (100) | 0 (0) | 1 (100) |  | 0 (0) | 1 (100) | 1 (100) |

**Table S8: Baseline age, laboratory and serology characteristics of patients by IgG level change between visit 1 and visit 2**

|  | Decline in IgG levels | | | | | | | | Incline in IgG levels | | | |
| --- | --- | --- | --- | --- | --- | --- | --- | --- | --- | --- | --- | --- |
|  | **N** | | **Mean (SD)** | | **Median (IQR)** | | **Min-Max** | | **N** | **Mean (SD)** | **Median (IQR)** | **Min-Max** |
| Age^*^ | 11 | 39.6 (9.09) | | 41 (30-44) | | 26-57 | | 52 | | 49 (11) | 49.5 (39.5-57.5) | 26-67 |
| Symptoms Duration | 11 | 2.73 (4.27) | | 1 (0-5) | | 0-14 | | 52 | | 3.29 (3.53) | 2.5 (0.5-4.5) | 0-14 |
| WBC | 11 | 5.54 (2.17) | | 5.1 (3.75-6.63) | | 2.94-10.1 | | 52 | | 6.11 (2.42) | 5.46 (4.75-7.03) | 2.64-17.6 |
| ALC | 11 | 2 (0.714) | | 2.22 (1.3-2.63) | | 0.91-2.98 | | 52 | | 1.57 (0.809) | 1.44 (1.08-1.98) | 0.21-4.5 |
| LDH | 11 | 212 (40.3) | | 204 (186-229) | | 164-302 | | 52 | | 230 (69.3) | 213 (182-265) | 126-478 |
| DDIMER | 10 | 0.747 (1.26) | | 0.27 (0.27-0.47) | | 0.27-4.3 | | 47 | | 0.374 (0.191) | 0.28 (0.27-0.43) | 0.02-1.11 |
| CPK | 11 | 356 (556) | | 112 (85-211) | | 44-1714 | | 52 | | 160 (116) | 117 (73.5-207) | 45-539 |
| Procalcitonin | 11 | 0.0445 (0.0324) | | 0.04 (0.02-0.06) | | 0.01-0.12 | | 52 | | 0.0567 (0.0723) | 0.03 (0.02-0.06) | 0.01-0.37 |
| Ferritin | 11 | 343 (435) | | 256 (104-310) | | 87-1621 | | 51 | | 393 (514) | 221 (133-449) | 16.5-2796 |
| CRP | 11 | 11 (16.9) | | 6 (1.7-14.1) | | 0.9-59.7 | | 52 | | 17.5 (26.4) | 6.15 (1.9-21.2) | 0.9-111 |
| QTC1 | 11 | 424 (21.6) | | 424 (408-442) | | 391-456 | | 52 | | 420 (20.7) | 425 (404-435) | 375-467 |
| QTC2 | 9 | 424 (18.1) | | 427 (416-436) | | 393-450 | | 49 | | 425 (19.7) | 424 (411-437) | 382-477 |
| QTC3 | 9 | 439 (22.9) | | 449 (425-454) | | 402-469 | | 49 | | 423 (20.8) | 421 (411-431) | 379-479 |
| Viral shedding (days) | 11 | 12.4 (4.97) | | 12 (9-17) | | 4-20 | | 50 | | 13.4 (6.46) | 13 (9-17) | 1-30 |
| Treatment Duration | 11 | 5.64 (2.11) | | 7 (5-7) | | 0-7 | | 52 | | 6.38 (1.4) | 7 (7-7) | 0-8 |
|  |  |  | |  | |  | |  | |  |  |  |
| Timing of first visit (days) | 11 | 17.8 (14.8) | | 15 (11-17) | | 2-60 | | 52 | | 15.3 (8.48) | 14.5 (10-19) | 3-57 |
| First visit IgG level | 11 | 92.5 (123) | | 39.1 (16.5-142) | | 7.9-401 | | 52 | | 65.1 (84.8) | 38 (12.6-73.3) | 3.5-401 |
| Timing of second visit (days) | 11 | 62 (27.1) | | 51 (45-94) | | 16-96 | | 52 | | 51.2 (20.6) | 50.5 (39-63.5) | 10-105 |
| Second visit IgG level | 11 | 56.3 (66.3) | | 38.1 (9.14-86) | | 6.61-224 | | 52 | | 126 (102) | 103 (56.7-149) | 7.45-401 |
| Timing of third visit (days) | 5 | 132 (21.4) | | 129 (121-129) | | 112-168 | | 22 | | 103 (37.1) | 119 (58-133) | 49-146 |
| Third visit IgG level | 5 | 53.4 (44.9) | | 50.4 (17.4-55.2) | | 17-127 | | 22 | | 117 (100) | 97.1 (46.8-138) | 26.7-401 |

^*^ significant difference between the two groups (p-value <0.05)

**Table S9: Baseline age, laboratory and serology characteristics of patients by IgG level change between visit 2 and visit 3**

|  | Decline in IgG levels | | | | | | | Incline in IgG levels | | | |
| --- | --- | --- | --- | --- | --- | --- | --- | --- | --- | --- | --- |
|  | **N** | | **Mean (SD)** | | **Median (IQR)** | **Min-Max** | | **N** | **Mean (SD)** | **Median (IQR)** | **Min-Max** |
| Age | 12 | 48.8 (10.2) | | 47 (42.5-55.5) | | 34-66 | 15 | | 47.9 (12.5) | 48 (38-58) | 26-65 |
| Symptoms Duration | 12 | 3 (2.63) | | 2 (1-4.5) | | 0-8 | 15 | | 3.27 (4.37) | 2 (0-3) | 0-14 |
| WBC | 12 | 6.62 (1.93) | | 6.6 (5.16-8.42) | | 3.35-9.34 | 15 | | 5.53 (1.18) | 5.24 (4.48-6.24) | 4.09-7.8 |
| ALC | 12 | 1.89 (0.841) | | 1.59 (1.38-2.34) | | 0.85-3.83 | 15 | | 1.63 (0.65) | 1.41 (1.14-2.13) | 0.65-2.95 |
| LDH | 12 | 248 (44.2) | | 243 (226-280) | | 172-314 | 15 | | 243 (87.6) | 194 (180-302) | 162-478 |
| DDIMER | 10 | 0.456 (0.258) | | 0.365 (0.29-0.55) | | 0.27-1.11 | 12 | | 0.413 (0.209) | 0.305 (0.27-0.47) | 0.27-0.93 |
| CPK | 12 | 193 (130) | | 131 (114-229) | | 76-539 | 15 | | 286 (420) | 98 (91-397) | 60-1714 |
| Procalcitonin | 12 | 0.0367 (0.0202) | | 0.025 (0.02-0.06) | | 0.02-0.07 | 15 | | 0.0947 (0.118) | 0.03 (0.02-0.18) | 0.01-0.37 |
| Ferritin | 12 | 248 (143) | | 184 (131-374) | | 113-489 | 15 | | 454 (450) | 271 (161-604) | 93-1621 |
| CRP | 12 | 10.2 (7.4) | | 10.8 (3.2-14.1) | | 0.9-23.8 | 15 | | 19.3 (29.5) | 6 (1.6-27.4) | 0.9-100 |
| QTC1 | 12 | 426 (21) | | 430 (409-438) | | 397-467 | 15 | | 422 (22.4) | 424 (400-433) | 386-458 |
| QTC2 | 12 | 431 (23) | | 430 (418-446) | | 395-477 | 15 | | 421 (17.8) | 422 (404-439) | 393-448 |
| QTC3 | 12 | 431 (23.4) | | 421 (415-447) | | 407-479 | 15 | | 422 (19) | 416 (407-441) | 392-453 |
| Viral shedding (days) ^*^ | 12 | 16.3 (5.58) | | 15.5 (12.5-19.5) | | 8-28 | 15 | | 11.5 (4.21) | 12 (9-13) | 2-20 |
| Treatment Duration | 12 | 6.08 (1.56) | | 7 (5-7) | | 2-7 | 15 | | 6.4 (1.06) | 7 (5-7) | 4-7 |
|  |  |  | |  | |  |  | |  |  |  |
| Timing of first visit (days) | 12 | 14.5 (7.23) | | 15 (9-20) | | 3-27 | 15 | | 16.7 (13.8) | 14 (9-17) | 3-60 |
| First visit IgG level | 12 | 74.2 (69.1) | | 52 (37.9-94.3) | | 3.5-249 | 15 | | 63.8 (114) | 19 (7.95-44.3) | 3.5-401 |
| Timing of second visit (days) | 12 | 43.9 (17.8) | | 48.5 (37-51.5) | | 10-77 | 15 | | 48.7 (27.3) | 48 (23-70) | 10-96 |
| Second visit IgG level | 12 | 140 (96.4) | | 117 (64.8-183) | | 29.7-334 | 15 | | 74.8 (77) | 58.4 (16.7-101) | 7.45-305 |
| Timing of third visit (days) | 12 | 115 (30.9) | | 121 (114-135) | | 49-146 | 15 | | 103 (40.3) | 121 (58-133) | 49-168 |
| Third visit IgG level | 12 | 81.1 (44) | | 80.6 (41.1-115) | | 26.7-153 | 15 | | 125 (120) | 81.2 (46.8-141) | 17-401 |

^*^ significant difference between the two groups (p-value <0.05)

**Table S10: Baseline age, laboratory and serology characteristics of patients by IgG level change between visit 1 and visit 3**

|  | Decline in IgG levels | | | | | | | Incline in IgG levels | | | |
| --- | --- | --- | --- | --- | --- | --- | --- | --- | --- | --- | --- |
|  | **N** | | **Mean (SD)** | | **Median (IQR)** | **Min-Max** | | **N** | **Mean (SD)** | **Median (IQR)** | **Min-Max** |
| Age | 8 | 44.6 (8.57) | | 43 (37.5-52) | | 34-58 | 19 | | 49.8 (12.2) | 49 (39-62) | 26-66 |
| Symptoms Duration | 8 | 2.63 (2.45) | | 1.5 (1-4.5) | | 0-7 | 19 | | 3.37 (4.07) | 2 (0-4) | 0-14 |
| WBC | 8 | 6.3 (2.15) | | 6.17 (4.58-8.12) | | 3.35-9.34 | 19 | | 5.9 (1.4) | 5.48 (4.99-6.9) | 4.09-9 |
| ALC | 8 | 1.78 (0.721) | | 1.93 (1.07-2.35) | | 0.85-2.7 | 19 | | 1.73 (0.764) | 1.51 (1.27-2.13) | 0.65-3.83 |
| LDH | 8 | 228 (44.2) | | 231 (185-272) | | 172-281 | 19 | | 253 (78.9) | 233 (185-307) | 162-478 |
| DDIMER | 6 | 0.305 (0.0497) | | 0.28 (0.27-0.34) | | 0.27-0.39 | 16 | | 0.48 (0.251) | 0.43 (0.27-0.58) | 0.27-1.11 |
| CPK | 8 | 127 (77.4) | | 106 (84.5-131) | | 63-309 | 19 | | 294 (374) | 195 (95-397) | 60-1714 |
| Procalcitonin | 8 | 0.0337 (0.0207) | | 0.02 (0.02-0.05) | | 0.02-0.07 | 19 | | 0.0837 (0.107) | 0.03 (0.02-0.08) | 0.01-0.37 |
| Ferritin | 8 | 283 (144) | | 309 (143-390) | | 93-489 | 19 | | 396 (417) | 199 (140-492) | 113-1621 |
| CRP | 8 | 15.2 (19.6) | | 9.1 (2.05-19.3) | | 0.9-59.7 | 19 | | 15.3 (24.3) | 6.1 (3.9-13.4) | 0.9-100 |
| QTC1 | 8 | 429 (19.6) | | 436 (413-441) | | 397-456 | 19 | | 422 (22.3) | 424 (399-432) | 386-467 |
| QTC2 | 8 | 430 (19.7) | | 434 (418-446) | | 395-453 | 19 | | 424 (21) | 422 (404-439) | 393-477 |
| QTC3^*^ | 8 | 441 (16.5) | | 444 (429-452) | | 415-465 | 19 | | 420 (20) | 415 (407-426) | 392-479 |
| Viral shedding (days) | 8 | 14.6 (3.62) | | 13.5 (12-18) | | 10-20 | 19 | | 13.2 (5.94) | 13 (9-15) | 2-28 |
| Treatment Duration | 8 | 5.88 (1.81) | | 7 (5-7) | | 2-7 | 19 | | 6.42 (1.02) | 7 (5-7) | 4-7 |
|  |  |  | |  | |  |  | |  |  |  |
| Timing of first visit (days) | 8 | 15.3 (7.5) | | 16 (10-20) | | 3-27 | 19 | | 15.9 (12.7) | 14 (8-18) | 3-60 |
| First visit IgG level | 8 | 126 (133) | | 62.8 (40.2-187) | | 24.5-401 | 19 | | 44.3 (63.6) | 19 (7.95-47.3) | 3.5-266 |
| Timing of second visit (days) | 8 | 52.5 (25.7) | | 50.5 (40-66.5) | | 10-96 | 19 | | 44.1 (22.4) | 47 (23-57) | 10-90 |
| Second visit IgG level | 8 | 108 (106) | | 75.9 (46.8-139) | | 8.58-334 | 19 | | 102 (86.6) | 85.5 (38.1-129) | 7.45-306 |
| Timing of third visit (days) | 8 | 120 (31) | | 129 (114-140) | | 49-146 | 19 | | 103 (38) | 119 (58-131) | 49-168 |
| Third visit IgG level | 8 | 69.3 (47) | | 48.9 (33.1-123) | | 17.4-127 | 19 | | 121 (107) | 88.2 (49.8-141) | 17-401 |

**Table S11:** Comparisonf of demographic and clinical characteristics of the patients having had one or two visits with those having had the three visits

|  | **Total** | **Visit 1 &/or 2 but not 3** | **All visits** | **p-value** |
| --- | --- | --- | --- | --- |
| N | 100 | 86 (76.1) | 27 (23.9) |  |
| Age groups |  |  |  | 0.925 |
| *20-29* | 9 (8.0) | 8 (9.3) | 1 (3.7) |  |
| *30-39* | 29 (25.7) | 22 (25.6) | 7 (25.9) |  |
| *40-49* | 28 (24.8) | 21 (24.4) | 7 (25.9) |  |
| *50-59* | 30 (26.5) | 23 (26.7) | 7 (25.9) |  |
| *60+* | 17 (15) | 12 (14) | 5 (18.5) |  |
| Gender |  |  |  | 0.739 |
| *Women* | 14 (12.4) | 10 (11.6) | 4 (14.8) |  |
| *Men* | 99 (87.6) | 76 (88.4) | 23 (85.2) |  |
| Nationality |  |  |  | 0.01 |
| *UAE national* | 42 (37.5) | 38 (44.2) | 4 (15.4) |  |
| *Non-national* | 70 (62.5) | 48 (55.8) | 22 (84.6) |  |
| Smoking |  |  |  | 0.563 |
| *No* | 110 (97.3) | 84 (97.7) | 26 (96.3) |  |
| *Yes* | 3 (2.7) | 2 (2.3) | 1 (3.7) |  |
| Symptoms |  |  |  | 0.886 |
| *No* | 24 (21.2) | 18 (20.9) | 6 (22.2) |  |
| *Yes* | 89 (78.8) | 68 (79.1) | 21 (77.8) |  |
| **Diagnostic tests** |  |  |  |  |
| PCR |  |  |  | 1.0 |
| *Negative* | 8 (7.1) | 6 (7) | 2 (7.4) |  |
| *Positive* | 105 (92.9) | 80 (93) | 25 (92.6) |  |
| CT |  |  |  | 1.0 |
| *Negative* | 7 (6.3) | 6 (7.1) | 1 (3.7) |  |
| *Positive* | 104 (93.7) | 78 (92.9) | 26 (96.3) |  |
| Case status |  |  |  | 0.549 |
| *Confirmed* | 96 (85.0) | 74 (86.0) | 22 (81.5) |  |
| *Suspected* | 17 (15.0) | 12 (14) | 5 (18.5) |  |
| **IgG measurements** |  |  |  |  |
| First IgG |  |  |  | 0.448 |
| *Negative* | 36 (31.9) | 29 (33.7) | 7 (25.9) |  |
| *Positive* | 77 (68.1) | 57 (66.3) | 20 (74.1) |  |
| Second IgG |  |  |  | 1.0 |
| *Negative* | 6 (9.5) | 3 (8.3) | 3 (11.1) |  |
| *Positive* | 57 (90.5) | 33 (91.7) | 24 (88.9) |  |
| Third IgG |  |  |  | NA |
| *Negative* | 0 (0) | 0 (0) | 0 (0) |  |
| *Positive* | 0 (0) | 0 (0) | 27 (100) |  |
| **Comorbidities** |  |  |  |  |
| DM |  |  |  | 0.585 |
| *No* | 88 (77.9) | 68 (79.1) | 20 (74.1) |  |
| *Yes* | 25 (22.1) | 18 (20.9) | 7 (25.9) |  |
| HTN |  |  |  | 0.249 |
| *No* | 81 (71.7) | 64 (74.4) | 17 (63) |  |
| *Yes* | 32 (28.3) | 22 (25.6) | 10 (37) |  |
| Heart disease |  |  |  | 0.444 |
| *No* | 104 (92) | 80 (93) | 24 (88.9) |  |
| *Yes* | 9 (8) | 6 (7) | 3 (11.1) |  |
| Renal disease |  |  |  | 0.592 |
| *No* | 108 (95.6) | 83 (96.5) | 25 (92.6) |  |
| *Yes* | 5 (4.4) | 3 (3.5) | 2 (7.4) |  |
| Respiratory disease |  |  |  | 1.0 |
| *No* | 109 (96.5) | 83 (96.5) | 26 (96.3) |  |
| *Yes* | 4 (3.5) | 3 (3.5) | 1 (3.7) |  |
| Immune disease |  |  |  | 1.0 |
| *No* | 112 (99.1) | 85 (98.8) | 27 (100) |  |
| *Yes* | 1 (0.9) | 1 (1.2) | 0 (0) |  |
| URTI |  |  |  | 0.82 |
| *No* | 81 (75.7) | 61 (76.3) | 20 (74.1) |  |
| *Yes* | 26 (24.3) | 19 (23.8) | 7 (25.9) |  |
| **Signs & symptoms** |  |  |  |  |
| Hypoxia |  |  |  | 1.0 |
| *No* | 101 (89.4) | 77 (89.5) | 24 (88.9) |  |
| *Yes* | 12 (10.6) | 9 (10.5) | 3 (11.1) |  |
| Fever |  |  |  | 0.785 |
| *No* | 46 (43) | 35 (43.8) | 11 (40.7) |  |
| *Yes* | 61 (57) | 45 (56.3) | 16 (59.3) |  |
| Cough |  |  |  | 0.871 |
| *No* | 49 (45.8) | 37 (46.3) | 12 (44.4) |  |
| *Yes* | 58 (54.2) | 43 (53.8) | 15 (55.6) |  |
| SOB |  |  |  | 0.734 |
| *No* | 94 (87.9) | 71 (88.8) | 23 (85.2) |  |
| *Yes* | 13 (12.1) | 9 (11.3) | 4 (14.8) |  |
| Fatigue |  |  |  | 0.497 |
| *No* | 73 (68.2) | 56 (70) | 17 (63) |  |
| *Yes* | 34 (31.8) | 24 (30) | 10 (37) |  |
| Loss of taste |  |  |  | 1.0 |
| *No* | 92 (86) | 69 (86.3) | 23 (85.2) |  |
| *Yes* | 15 (14) | 11 (13.8) | 4 (14.8) |  |
| Loss of smell |  |  |  | 0.756 |
| *No* | 92 (86) | 68 (85) | 24 (88.9) |  |
| *Yes* | 15 (14) | 12 (15) | 3 (11.1) |  |
| Sore throat |  |  |  | 0.771 |
| *No* | 81 (75.7) | 60 (75) | 21 (77.8) |  |
| *Yes* | 26 (24.3) | 20 (25) | 6 (22.2) |  |
| Nausea & vomiting |  |  |  | 0.444 |
| *No* | 98 (91.6) | 72 (90) | 26 (96.3) |  |
| *Yes* | 9 (8.4) | 8 (10) | 1 (3.7) |  |
| **Management/Treatment** |  |  |  |  |
| ICU/HDU |  |  |  | 1.0 |
| *No* | 109 (96.5) | 83 (96.5) | 26 (96.3) |  |
| *Yes* | 4 (3.5) | 3 (3.5) | 1 (3.7) |  |
| Hydroxychloroquine |  |  |  | 0.002 |
| *No* | 22 (19.5) | 22 (25.6) | 0 (0) |  |
| *Yes* | 91 (80.5) | 64 (74.4) | 27 (100) |  |
| Favipiravir |  |  |  | 0.318 |
| *No* | 47 (41.6) | 38 (44.2) | 9 (33.3) |  |
| *Yes* | 66 (58.4) | 48 (55.8) | 18 (66.7) |  |
| Kaletra (Lopinavir and ritonavir) |  |  |  | 1.0 |
| *No* | 107 (94.7) | 81 (94.2) | 26 (96.3) |  |
| *Yes* | 6 (5.3) | 5 (5.8) | 1 (3.7) |  |

Figure S1 IgG levels during the three visits, among the 27 having a complete follow-up

Figure S2 IgG levels during the three visits and according to the month, among asymptomatic/ symptomatic patients
