# Supplementary material for: Longitudinal changes in IgG levels among COVID-19 recovered patients: A prospective cohort study
Source: PLoS One. 2021 Jun 11;16(6):e0251159. doi: 10.1371/journal.pone.0251159 (PMC8195379; doi:10.1371/journal.pone.0251159)
Supplement: Attachment — Submitted filename: Response to reviewers_14APR2021.docx [file pone.0251159.s002.docx]

**Response to reviewers :**

Dear editor,

We thank you and the reviewers for their comments. We have clarified some points and made the changes as suggested by reviewer1 and have taken into account the editorial office comments. Please see below our point-by-point responses.

We hope that this revised version is now suitable for publication in PLOS ONE.

Best regards,

Journal Requirements:

When submitting your revision, we need you to address these additional requirements.

1. Please review your reference list to ensure that it is complete and correct. If you have cited papers that have been retracted, please include the rationale for doing so in the manuscript text, or remove these references and replace them with relevant current references. Any changes to the reference list should be mentioned in the rebuttal letter that accompanies your revised manuscript. If you need to cite a retracted article, indicate the article’s retracted status in the References list and also include a citation and full reference for the retraction notice.

We have updated the references : 4 pre-print have been published since our submission, we have updated the references. 2 pre-prints remained unpublished. All atucles included in the references sections were passed through the retraction database (<http://retractiondatabase.org>) and no paper cited have been retracted

2. Please ensure that your manuscript meets PLOS ONE's style requirements, including those for file naming. The PLOS ONE style templates can be found at <https://journals.plos.org/plosone/s/file?id=wjVg/PLOSOne_formatting_sample_main_body.pdf> and https://journals.plos.org/plosone/s/file?id=ba62/PLOSOne_formatting_sample_title_authors_affiliations.pdf

 We have ensured that our manuscript meets PLOS ONE’s style requirements, particularly with title, figure, and heading style.

3. PLOS requires an ORCID iD for the corresponding author in Editorial Manager on papers submitted after December 6th, 2016. Please ensure that you have an ORCID iD and that it is validated in Editorial Manager. To do this, go to ‘Update my Information’ (in the upper left-hand corner of the main menu), and click on the Fetch/Validate link next to the ORCID field. This will take you to the ORCID site and allow you to create a new iD or authenticate a pre-existing iD in Editorial Manager. Please see the following video for instructions on linking an ORCID iD to your Editorial Manager account: <https://www.youtube.com/watch?v=_xcclfuvtxQ>

We have linked the first author with his ORCID iD (0000-0001-9840-2339) :

Additional Editor Comments:

Overall, the manuscript is technically sound and the data support the conclusions. However, all the points raised by Reviewer 1 need to be addressed. Points 1 and 2 are especially important to supporting the claims that a cutoff of 15 AU/ml is appropriate and the study includes a multi-ethnic population.

We agree that the points 1 and 2 raised by the Reviewer are crucial and therefore we have add more details in the manuscript (detailed response below in the Response to the Reviewer 1) :

1) the cutoff of 15 AU/ml is the one recommended by the manufacturer, and a sensitivity/specificity analysis confirmed its adequacity (see more detailed response below).
2) We have included in the description of our population the percentage of each nationality within the “non national Emirates” group : the non nationals breakdown is as follows: 47 from Asia ,14 from Middle East , 4 from South America and 5 with no data on nationality.

We have also corrected the Table 1 as the table was describing only 112 individuals instead of 113.

In addition, the authors should also:

1. Discuss whether they expect the observed longitudinal changes to be the same for each IgG subclass (IgG1, G2, G3, G4) or different by subclass

The IgG subclasses were not available to be tested at the time when we conducted our study. We declare this as a limitation of our study. We don’t know if the subclasses measurement would have the same trend and the current literature on the measurement of IgG subclasses is quite inconclusive. Furthermore, the literature mostly studies IgG, regardless of the subclasses.

2. Explicitly describe the limitations of the study (only focuses on IgG and not other isotypes, only looks at IgG antibodies against S1 & S2 and not other antigens etc).

We have added in the discussion section describing this limitation : “One of the limitations of our study is that we did not have data on IgG subclasses. Also, our IgG antibodies focused on S1 and S2 antigens only and no other isotypes. However, S protein is the main protein used as a target in COVID-19 vaccines and thus of very high interest.”

Reviewers' comments:

Reviewer's Responses to Questions

**Comments to the Author**

1. Is the manuscript technically sound, and do the data support the conclusions?

The manuscript must describe a technically sound piece of scientific research with data that supports the conclusions. Experiments must have been conducted rigorously, with appropriate controls, replication, and sample sizes. The conclusions must be drawn appropriately based on the data presented.

Reviewer #1: Yes

2. Has the statistical analysis been performed appropriately and rigorously?

Reviewer #1: Yes

3. Have the authors made all data underlying the findings in their manuscript fully available?

The [PLOS Data policy](http://www.plosone.org/static/policies.action#sharing) requires authors to make all data underlying the findings described in their manuscript fully available without restriction, with rare exception (please refer to the Data Availability Statement in the manuscript PDF file). The data should be provided as part of the manuscript or its supporting information, or deposited to a public repository. For example, in addition to summary statistics, the data points behind means, medians and variance measures should be available. If there are restrictions on publicly sharing data—e.g. participant privacy or use of data from a third party—those must be specified.

Reviewer #1: Yes

4. Is the manuscript presented in an intelligible fashion and written in standard English?

PLOS ONE does not copyedit accepted manuscripts, so the language in submitted articles must be clear, correct, and unambiguous. Any typographical or grammatical errors should be corrected at revision, so please note any specific errors here.

Reviewer #1: Yes

5. Review Comments to the Author

Please use the space provided to explain your answers to the questions above. You may also include additional comments for the author, including concerns about dual publication, research ethics, or publication ethics. (Please upload your review as an attachment if it exceeds 20,000 characters)

Reviewer #1: The manuscript by Alzaabi et al describes a prospective cohort study of the longitudinal antibody response in adult COVID-19 patients in the UAE over a period of 6 months. Both symptomatic and asymptomatic patients were included within the cohort, and the many clinical parameters were recorded for each patient. The IgG response against SARS-CoV-2 S1 and S2 were determined using an FDA-EUA chemiluminescent immunoassay (CLIA) platform, and the authors find that across patients who returned for all three visits spanning 6 months, peak antibody titers against S1/S2 were observed at the second visit (approximately at month 3 post-symptom onset), and reduced slightly, but not significantly by the third visit (6 months post-symptom onset). They find that of those who returned for visit 3, all patients had developed humoral immunity.
Their data is consistent with reports from other countries regarding the longevity of the humoral immune response up to 6 months after symptom onset. The authors perform subsequent analysis between IgG seropositivity and clinical measures and find that seropositive patients at visit 1 had increased levels of LDH and ferratin compared with seronegative patients. The authors note trends in the data, where a majority of subjects have increasing IgG responses between visits, and maintained through day 120, and some patients show decreasing trends between visits. They show that symptomatic and asymptomatic patients do not differ in levels of IgG.
The authors present their study in a coherent and logical manner, and the inclusion of a comprehensive discussion of similar studies is welcome and places the author’s data in context. Their data set contributes to our understanding of long-term humoral immunity and against SARS-CoV-2 in populations around the globe.

Major points:
1. Were any control samples (pre-pandemic or non-COVID) included in the IgG analysis and used to establish the baseline cutoff of 15 AU/ml?

We have added a complete document “Liaison SARS CoV-2 S1S2 IgG Method Performance Verification” in the supplementary material. More specifically :

One hundred presumed SARS-CoV-2 negative samples collected before the COVID19 outbreak (80 fresh frozen plasma (FFP) samples from blood donors, 10 stored HIV positive samples, and 10 stored Anti-nuclear autoantibodies (ANA) positive patient’s samples) were tested resulting in 99.25% clinical specificity (95% CI: 98.5% – 100%). The following table shows the results:

|  | LIAISON® SARS-CoV-2 S1/S2 IgG | | | Total | Specificity (Wilson 95% CI) |  |
| --- | --- | --- | --- | --- | --- | --- |
|  | < 12 AU/mL | 12-15 AU/mL | ≥ 15 AU/mL |  |  | |
| Pre pandemic FFP samples | 78 | 1 | 1 | 80 | 98.5% (97.5% - 99.2%) | |
| Pre pandemic HIV positive samples | 10 | 0 | 0 | 10 | 100 % | |
| Pre pandemic ANA positive samples | 10 | 0 | 0 | 10 | 100% | |

The sensitivity was determined by investigating 50 patient’s samples collected over the course of time. Infection with SARS-CoV-2 was confirmed by RT-PCR test at the time of the diagnosis. The LIAISON® SARS-CoV-2 S1/S2 IgG test was performed on samples collected at the time of admission and thereafter up.

The diagnostic sensitivity was evaluated in three groups, i.e. the early samples (≤ 5 days after diagnosis), the samples between 5 and 15 days after diagnosis, and the later samples (> 15 days after diagnosis).

|  | LIAISON® SARS-CoV-2 S1/S2 IgG | | | Total | Sensitivity (Wilson 95% CI) |
| --- | --- | --- | --- | --- | --- |
|  | < 12 AU/mL | 12-15 AU/mL | ≥ 15 AU/mL |  |  |
| ≤ 5 days | 11 | 1 | 4 | 16 | 24.9% (14.0%-39.4%) |
| 5-15 days | 1 | 1 | 12 | 14 | 90.2% (79.2%-95.8%) |
| ≥ 15 days | 0 | 1 | 19 | 20 | 97.3% (86.8%-99.5%) |

2. The authors mention that the patients represent a multi-ethnic and global population but only stratify the patients into Emerati national vs non-national. While the authors note that there were no differences in IgG across ethnicity/race, does that analysis refer to Emerati national vs non-national or to a more thorough breakdown and analysis of ethnicity/race?

The analysis refers indeed to Emerati national vs non-national, as it is stated in the last paragraph of the result section “The patients with three IgG level measurements did not differ from those having only one IgG measurement, except regarding nationality: the patients with complete follow-up were much more Non-national”

We have included in the description of our population the number of participants of each nationality within the “non national Emirates” group to detail the “multi ethnic settings of our population”. However, we were not able to stratify our results as we did not have enough power.

3. It would be helpful if the authors could also plot the individual data points for the figures 2 and 3 in an overlay of the IQR plot, showing the variation across patients at each time point.

We agree with the reviewer point that showing simultaneously the data points improves the plots. We have changed the plots according to this comment.

4. Figure 4 is a little confusing to me in the way it is currently presented, and although I understand the point that the authors are making with both Figure 4 and Table 3, I wonder if the maybe color coding each visit separately would help the readers correlate Figure 4 with Table 3 better. Another possibility may be to present all three visits on the same graph for all patients to give the readers a sense of which subjects responses are increasing/decreasing across the 6 months, or if there are some patients who are initially increasing, and then decrease. Also, it seems like the order of the graphs might make sense to switch to the 4th (Increase/Decrease in IgG response v1 vs v3) and 3rd graphs (Increase/Decrease over 90 days) to flow better with the text.

We agree with the reviewer that Figure 4 may be a little bit confusing. We have changed the color (one for visit1, one for visit2 and one for visit3). We have also changed the order of the graph, as suggested.

5. The number of clinical parameters that the authors collect on each patient represents a rich dataset for their COVID patients – does the anti-spike IgG response correlate with any clinical parameters in a regression analysis? A multivariate analysis approach of the data set may indicate parameters such as duration of viral shedding, treatments, or disease severity as drivers of IgG induction and maintenance. Along those lines, as the authors are able to divide patients into those whose titers increases vs those whose titers decrease, another interesting analysis would be if any of the clinical parameters could be used to predict increasing or decaying antibody responses. These analyses would help increase the relevance of the author’s work.

We agree with the reviewer that it would have been very interesting to look deeper in the individual factor associated to IgG longitudinal changes. However, we have not enough power to do such analysis.

We ran univariate analyses to test the association between several factors (age, nationality, symptoms, presence of comorbidity, viral shedding, treatment duration, and Biochemical parameters) and an increase in IgG response from first to second visit. Only age, nationality and QTC3 were statistically significant and when adjusting on age, no more statistically significant association was found. This lack of result could be due to the small sample size, but no conclusion can be made with our data. We have added a sentence to state our results (in the method and in the results section).

Minor comments:
1. Open parentheses missing in line 3

We have corrected this.
2. Line 22: suggest changing to “COVID-19 patients”.

We have followed your suggestion.
3. Please include the dilutions (if any) of sera used in serology testing in the Methods.

We did not apply any dilution protocol for samples with results above assay range (>400 AU/mL), we reported it as it is.

4. Figure 4 is missing the labels for the graphs (a, b, c, etc.), and are labeled incorrectly as 2a, 2b, 2c etc. in the legend.
We have corrected this.
Reviewer: Bronwyn M. Gunn, PhD.
